# Quality of life in primary caregivers of patients in peritoneal dialysis and hemodialysis

**DOI:** 10.1590/2175-8239-JBN-2020-0229

**Published:** 2021-06-04

**Authors:** Joel Monárrez-Espino, José Alberto Delgado-Valles, Gastón Ramírez-García

**Affiliations:** 1 Hospital Christus Muguerza, Departamento de Investigación, Chihuahua, México.; 2 Universidad de Monterrey, San Pedro Garza García, México.; 3 Claustro Universitario de Chihuahua, Grupo de Investigación en Salud Pública. Chihuahua, México.

**Keywords:** Renal Dialysis, Quality of Life, Mexico, Peritoneal Dialysis, Caregivers, Diálise Renal, Qualidade de Vida, México, Diálise Peritoneal, Cuidadores

## Abstract

**Background:**

Peritoneal dialysis (PD) is gaining track as an efficient/affordable therapy in poor settings. Yet, there is little data regarding differences in quality of life (QoL) of primary caregivers (PCG) of patients in PD and hemodialysis (HD).

**Aim:**

To compare the QoL of PCG of patients in PD and HD from an upper middle-income population in a Mexican city.

**Methods:**

Cross-sectional study was carried out with PCG of patients in PD (n=42) and HD (n=95) from 4 hospitals (response rate=70.2%). The SF 36-item QoL questionnaire, the Zarit burden interview, and the Goldberg anxiety/depression scale were used. Mean normalized scores for each QoL domain were compared by dialysis type. Adjusted odds were computed using logistic regression to determine the probability of low QoL (<70% of maximum possible score resulting from the added scores of the 8 dimensions).

**Results:**

The PD group had higher mean scores for emotional role functioning (+10.6; p=0.04), physical functioning (+9.2; p=0.002), bodily pain (+9.2; p=0.07), social functioning (+5.7; p=0.25), and mental health (+1.3; p=0.71); the HD group had higher scores for physical role functioning (+7.9, p=0.14), general health perception (+6.1; p=0.05), and vitality (+3.3; p=0.36). A non-significant OR was seen in multivariate regression (1.51; 95% CI 0.43-5.31). Zarit scores were similar, but workload levels were lower in the PD group (medium/high: PD 7.2%, HD 14.8%). Anxiety (HD 50.5%, PD 19%; p<0.01) and depression (HD 49.5%, PD 16.7%; p<0.01) were also lower in the PD group.

**Conclusion:**

Adjusted analysis showed no differences in the probability of low QoL between the groups. These findings add to the value of PD, and strengthen its importance in resource-limited settings.

## Resumo

**Histórico:**

A diálise peritoneal (DP) vem ganhando terreno como terapia eficiente/acessível em ambientes pobres. Contudo, há poucos dados sobre diferenças na qualidade de vida (QV) dos cuidadores primários (CP) de pacientes em DP e hemodiálise (HD).

**Objetivo:**

Comparar QV dos CP de pacientes em DP e HD de uma cidade mexicana de renda média.

**Métodos:**

Estudo transversal com CP de pacientes em DP (n=42) e HD (n=95) de 4 hospitais (taxa resposta=70,2%). Aplicou-se o questionário QV-36 itens, entrevista de sobrecarga de Zarit e escala de ansiedade/depressão Goldberg. Escores médios normalizados para cada domínio de QV foram comparados por tipo de diálise. Probabilidades ajustadas foram calculadas usando regressão logística para determinar a probabilidade de baixa QV (<70% da pontuação máxima possível resultante das pontuações adicionadas das 8 dimensões).

**Resultados:**

O grupo DP apresentou escores médios mais altos para aspectos emocionais (+10,6; p=0,04), capacidade funcional (+9,2; p=0,002), dor (+9,2; p=0,07), aspectos sociais (+5,7; p=0,25), saúde mental (+1,3; p=0,71); o grupo HD teve pontuação maior para aspectos físicos (+7,9, p=0,14), estado geral de saúde (+6,1; p=0,05), vitalidade (+3,3; p=0,36). Uma probabilidade não significativa foi observada na regressão multivariada (OR=0,66; 95% IC 0,18-2,31). Os escores de Zarit foram semelhantes, mas os níveis da sobrecarga foram menores na DP (médio/alto: DP 7,2%, HD 14,8%). Ansiedade (50,5% vs 19%; p<0,01) e depressão (49,5% vs 16,7%; p<0,01) foram menores na DP.

**Conclusão:**

O risco de baixa QV entre grupos não foi diferente na análise ajustada. Estes achados fortalecem a importância da DP em ambientes com recursos limitados.

## Introduction

Globally, an estimated 5-10 million people die every year from chronic kidney disease^1^. This is an irreversible illness that progressively erodes the patients’ health and quality of life (QoL). In Mexico, incidence and prevalence have been increasing steadily^2^^,^ 3 to the point that, soon, nearly 200 thousand individuals will require renal replacement therapy^4^. Currently, peritoneal dialysis (PD) and hemodialysis (HD) are the two main alternatives available for most patients^5^.

While HD is by far the most common modality worldwide, especially in developed countries^6^, PD is becoming a major alternative in low- and middle-income settings^5^^,^ 7 ^-^ 10, as it has shown to be the most economically efficient dialysis modality^11^^-^ 12, in fact, two out of three patients who receive PD live in developing nations^13^.

Mexico is the leading country in the world using PD, partly due to the costs involved (PD can be 44-78% cheaper than HD)^14^^-^ 16. According to the Mexican Institute of Social Security, the largest public provider of health services in Mexico, 77% of the patients in this institution were treated with PD and 23% with HD in 2015^15^.

Patients in dialysis have to modify their lifestyle in terms of nutrition, daily habits, mental health, physical activity, and social/family relations due to the restrictions linked to the procedure itself^17^^,^ 18. In most developing countries, patients in dialysis rely on a primary caregiver (PCG) for their care, usually the spouse or an adult child^19^.PCGs are individuals who voluntarily assume responsibility for an ill patient in its broader sense, usually without financial remuneration^18^.

It has been reported that PCGs need appropriate knowledge, skills, and guidance to provide adequate care to patients in dialysis^20^^-^ 22, as they require comprehensive therapeutic measures, especially those in PD, which is generally performed at home^22^.

Caregiving is regarded as a chronic stressor due to the emotional burden, and the persistent and often physically demanding activities; the logistics and management of symptoms and treatment associated with the dialysis process (e.g. transportation to the dialysis unit, medical appointments, diet control, personal hygiene support, etc.) can have an important impact on the caregiver’s QoL^18^^,^ 21 ^,^ 23.

The PCGs’ work overload can also affect their QoL. This relates to factors such as the main illness leading to the end-stage renal disease (ESRD), the ability and existing resources available to take care of patients, and the concomitant morbidities^18^^,^ 20 ^,^ 24. The daily and long-term care of a sick family member can also entail health risks for caregivers, especially when the responsibility falls on a single person.

The fact that caregivers enter a process of physical and emotional erosion, derived from the implications of treatment and permanent care at home for prolonged periods of time, added to the economic hardships and family difficulties associated with the existence of this condition that frequently prevents PCGs from living in a conventional family, social, and work life^18^^,^ 25 ^,^ 26.

As a result, these modifications translate into lifestyle changes of PCGs^27^^,^ 28. These changes, however, seem to differ between PCGs of patients in HD and PD. Being a home-based modality, PD in low- and middle-income countries can have some advantages for PCGs compared with HD, including lower transportation and other costs associated with hospital visits, greater convenience as patients can be dialyzed at home avoiding the 5-6 h required for each hospital visit, and increased autonomy and flexibility as patients are not dependent on a hospital^5^^,^ 9 ^,^ 11 ^,^ 12. On the other hand, PD PCGs require more training, might need to deal with more complications, and have to take care of the logistics involved with the procedure^29^.

In a systematic review on QoL among PCGs published in early 2019, it was concluded that QoL was “comparable” between dialysis types^19^. Yet, such conclusion was based on only three studies, two that used data collected nearly 20 years ago, one showing differences in some QoL dimensions^30^, and the other reporting no differences^27^; the third showed a lower level of burden in PCGs of HD patients compared with those in PD (13 vs. 35%)^31^. However, two recent studies from Turkey and India, not included in that review, reported contrary results, showing a higher burden for caregivers of HD patients^32^^,^ 33. While this topic still remains contentious, based on the available evidence from countries relatively similar to Mexico, we hypothesized that PCGs of PD patients would have at least the same burden or possibly lower burden compared with that of those caring for HD patients. Therefore, this study was done to shed light on this issue by comparing the QoL of PCGs of patients in HD and PD from an upper middle-income city of northern Mexico.

The research proposal was revised and approved by the Ethics and Research Committee at Christus Muguerza Hospital Chihuahua (CEI-HCMP-03042018-1). Informed consent was obtained from all participant caregivers.

## Material and Methods

### Study Design

This was a cross-sectional multicenter study with PCGs of patients in PD and HD carried out between May and October 2019.

### Study Population And Setting

Patients and PCGs were male and female adult residents of the northern Mexican city of Chihuahua, the capital of the homonymous State that has a high prevalence of patients with ESRD^34^. The city is industrialized, and ranks high in human and social development among cities in Mexico.

Eight hospitals provide dialysis services to patients with ESRD in this city of nearly one million inhabitants. This study was carried out in the four hospitals that provided permission to collect data from patients and PCGs.

Patients were asked if they had a main caregiver responsible for helping them withstand their health condition and dialysis, but the degree of assistance provided by the caregiver was not assessed. Yet, the vast majority of the participant PCGs accompanied their patient to the medical appointments and reported duties associated with the illness and dialysis process.

### Inclusion Criteria

Eligible patients had to be dialyzed at least within one month prior to the interview. PCGs of patients from three out of the five public hospitals that provide dialysis services in Chihuahua were included (Hospital of the Institute of Services and Social Security for State Workers, the State Civil Pensions Hospital, and the General Hospital). Also, PCGs from one of the three private hospitals that offer dialysis were included (Christus Muguerza Hospital). PCGs had to consent and be able to answer questionnaires through face-to-face interviews.

### Sampling

From the 207 eligible patients within the four hospitals sampled, 195 had a PCG (94.2%); from these, 137 were surveyed: 24 PCGs refused to participate, 31 could not be contacted, and three agreed but did not attend the interview. Thus, the participation rate for PCGs was 70.2%.

### Data Collection And Measurement Instruments

Data was collected in 2019 by seven trained and standardized field workers. PCGs of both HD and PD patients were interviewed in a medical office. The average interview duration was 30 min for each PCG.

Four instruments were administered:

1. ***General questionnaire:*** It was used to collect sociodemographic (i.e. sex, age, civil status, schooling years, religion, occupation, number of dependents, and hospital of care), anthropometric (i.e. weight and height), and clinical data (i.e. comorbidities, medical treatments, surgeries, and smoking/alcohol/drug history). It also included some questions related to the patient care (e.g. type of relationship -kinship- with the patient, duration of care, and approximate amount of money spent per month for the care of his/her patient).
2. ***Short form 36-item QoL questionnaire:*** It consists of 36 items that fit into 8 QoL domains: physical functioning, physical role, body pain, general health, vitality, social function, emotional role, and mental health^35^. The number of items per domain varies from two to ten. Depending on the item, the score can range from 1 to 3 to 1 to 6 points. The total raw score for each domain is then normalized so that the final scale ranges from 0 to 100^35^. The internal consistency is >0.7^36^.
3. ***Zarit burden interview:*** It consists of 22 items that measure the burden perceived by the caregiver using a Likert scale ranging from 0 (never) to 4 (always). Adding the 22 scores, a unique burden index is obtained with a score ranging from 0 to 88 points. The total score is then grouped as: without (≤21), light (22-46), medium (47-55), and severe (≥56 points) burden. Cronbach’s alpha for the validation study in Mexico was 0.84 with a model fit with values ≥0,90^37^.
4. ***Goldberg anxiety and depression scale:*** This screening tool consists of scales for anxiety and depression with 9 items each. Responses are dichotomous. An independent score is totalized for each scale. The patient is questioned about whether he/she has presented any of the relevant symptoms; those lasting <2 weeks or of mild intensity are not scored. The cut-off point for anxiety and depression was **≥**4 and **≥**2, respectively. An adequate internal and external validity has been reported; correlation coefficient with the Hamilton Depression Scale is 0.74^38^.

### Statistical Analysis

Frequencies of selected sociodemographic characteristics of PCGs of patients in HD and PD were tabulated and compared using Pearson Chi-square and Fisher’s tests. Mean normalized scores and standard deviation (s.d.) for the eight QoL domains were computed and compared by dialysis type using Student’s t-tests. Means were also depicted using a radial graph. Also, Zarit and Golberg scores of PCGs of patients in HD and PD were compared using parametric and non-parametric statistics.

Crude and adjusted odds ratios (OR) with 95% confidence intervals (CI) were computed from binary logistic regression for the probability that PCGs had a low QoL, conventionally defined as less than 70% of the maximum possible score resulting from the added normalized scores of the eight dimensions. This cut-off was deemed as a fair definition for insufficient QoL, even though other authors have even proposed a more stringent cut-off of <60% for a similar population group^39^. All variables collected from the general questionnaire, Zarit interview, and Goldberg scale were tested in bivariate analyses (QoL as continuous dependent variable) using parametric (Student’s t-tests and ANOVA) and non-parametric (Mann-Whitney and Kruskal-Wallis tests) statistics. Variables considered potential confounders^40^, both conceptually (i.e. covariates that are related to both the exposure and outcome) and statistically (p-value ≤0.10) were entered in the full model, but the final adjusted model included only statistically significant (p<0.05) variables. The model’s goodness-of-fit was assessed using the Hosmer & Lemeshow Chi-square test with a non-significant p-value indicating a good fit. The Nagelkerke R^2^ statistic was used to determine the percentage of prediction of the model. All data was entered and analyzed in SPSS^®^ v.24.

## Results

Table 1 compares sociodemographic data for PCGs of patients in HD and PD. There was a higher proportion of female PCGs in both dialysis groups (HD 80%, PD 92.9%). However, the proportion of male PCGs was higher in HD patients (20%) compared with PD patients (7.1%). No statistical differences were observed in all other variables tested. Regardless of dialysis group, most PCGs were aged 41-60 years (HD 56.8, PD 42.9), married (HD 80%, PD 73.8%), had more than 12 years of formal education (HD 54.7%, PD 62.9%), and half were married or cohabiting with the patient (HD 53.7%, PD 50%).

**Table 1:** Selected sociodemographic characteristics of caregivers of patients in hemodialysis and peritoneal dialysis

Variable	Category	n (%)		
		Hemodialysis	p-value*	Peritoneal dialysis
		
Sex	Male	19 (20.0)	0.07	3 (7.1)
	Female	76 (80.0)		39 (92.9)
Age group in years	15-40	18 (18.9)	0.28	12 (28.6)
	41-60	54 (56.8)		18 (42.9)
	>60	23 (24.2)		12 (28.6)
Civil status	Married or cohabiting	76 (80.0)	0.25	31 (73.8)
	Single or divorced	16 (16.8)		11 (26.2)
	Widowed	3 (3.2)		0 (0.0)
Formal schooling (years)	1-6 (primary)	9 (9.5)	0.52	3 (7.1)
	7-9 (secondary)	15 (15.8)		9 (21.4)
	10-12 (high school)	17 (17.9)		4 (9.5)
	>12 (college or more)	52 (54.7)		26 (62.9)
	Unknown	2 (2.1)		0 (0.0)
Religion	Catholic	84 (88.4)	0.54	35 (83.3)
	Protestant	6 (6.3)		5 (11.9)
	None or other	5 (5.3)		2 (4.8)
Relationship to patient	Spouse/partner	51 (53.7)	0.24	21 (50.0)
	Daughter/son	28 (29.5)		16 (38.1)
	Parent/sibling	10 (10.5)		1 (2.4)
	Others (relative, nurse)	4 (4.2)		4 (9.5)
	Unknown	2 (2.1)		0 (0.0)
Total		95		42

* Pearson Chi-square and Fisher's tests were used.

Table 2 compares mean normalized scores for the eight QoL domains between HD and PD PCGs. The mean total normalized score was slightly higher in the PD compared with the HD group, but the difference did not reach statistical significance (PD 606, HD 587; p=0.37). However, PCGs of patients in PD had higher scores for emotional role functioning (+10.6; p=0.04), physical function (+9.2; p=0.002), bodily pain (+9.2; p=0.07), social functioning (+5.7; p=0.25), and mental health (+1.3; p=0.71). Conversely, PCGs of patients in HD had somewhat higher mean scores for physical role functioning (+7.9, p=0.14), general health (+6.1; p=0.05), and vitality (+3.3; p=0.36).

**Table 2:** Mean normalized scores and standard deviation (s.d.) for the eight domains of the Quality of Life SF-36 between caregivers of patients in hemodialysis and peritoneal dialysis

Domain	Mean±s.d.		
	Hemodialysis n=95	p-value*	Peritoneal dialysis n=42
Physical functioning	82.1±22.4	0.00	91.3±11.1
Physical role functioning	74.1±30.4	0.14	66.2±24.6
Bodily pain	71.2±27.3	0.07	80.4±27.4
General health	66.4±22.0	0.05	60.3±14.2
Vitality	64.9±24.9	0.36	61.6±17.2
Social functioning	77.7±27.7	0.25	83.4±23.7
Emotional role functioning	78.2±30.7	0.04	88.8±20.7
Mental health	73.1±25.8	0.71	74.4±14.1
Total score^1^	587.9±156	0.37	606.6±88.8

* Student t-tests were used

1 Computed by adding the normalized individual scores for the 8 dimensions.

**Figure 1:**
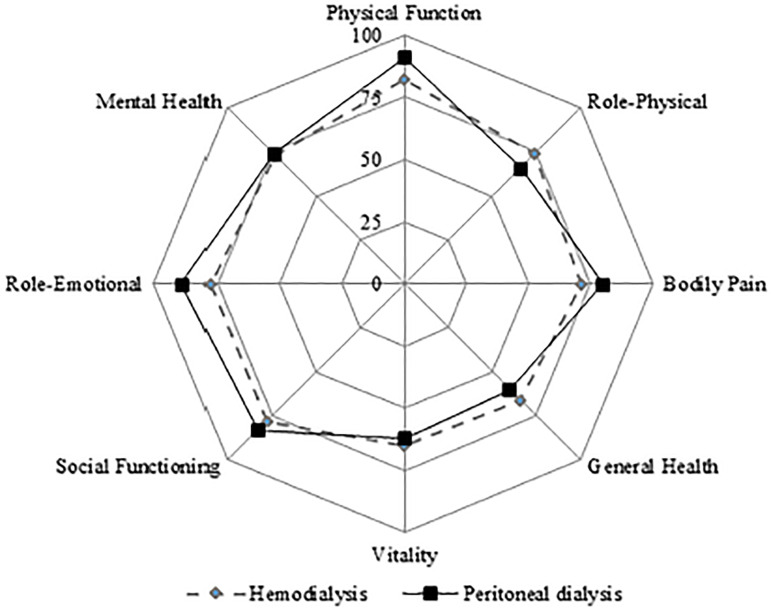
Average normalized scores for the eight QoL domains studied for primary caregivers of patients in hemodialysis (n=95) and peritoneal dialysis (n=42).

The mean normalized scores for the eight QoL domains for PCGs of patients in HD (n=95) and PD (n=42) is graphically presented in Figure 1.

Table 3 compares work overload levels and prevalence of anxiety and depression between PCG of patients in HD and PD. Both mean (HD 23.6, PH 22.8; p=0.77) and median (HD 18, PH 21.5; p=0.85) Zarit scores were relatively similar, yet, workload levels tended to be lower in PCGs of patients in PD (medium + high load: HD 14.8%, PD 7.2%). The proportion of anxiety (HD 50.5%, PD 19%; p<0.01) and depression (HD 49.5%, PD 16.7%; p<0.01) was also considerably lower among PCGs of patients in PD according to the Goldberg scale.

**Table 3:** Comparison of the Zarit (work overload) and Golberg (anxiety and depression) scales between caregivers of patients in hemodialysis and peritoneal dialysis

Scale used	Hemodialysis n=95	p-value*	Peritoneal dialysis n=42
Zarit (work overload)			
Mean ± standard deviation	23.6±17.4	0.77	22.8±14.2
Median (min-max)	18.0 (0-67)	0.85	21.5 (0-75)
Light load: 22-46 points, n (%)	30.5%	0.25	45.2%
Medium load: 47-55 points, n (%)	9.5%		2.4%
Severe load: >56 points, n (%)	5.3%		4.8%
Goldberg			
Anxiety: ≥2/9 items, n (%)	50.5%	0.00	19.0%
Depression: ≥1/9 items, n (%)	49.5%	0.00	16.7%

* Student t and Mann-Whitney tests were used for continuous data, and Pearson Chi-square, and Fisher tests were used for nominal data.

The logistic regression to determine the probability of low QoL among PCGs by type of dialysis of the patient is presented in Table 4. Crude OR for HD compared with PD was 1.86 (95% CI 0.79-4.36). PCGs’ age group, care time in months, work overload, anxiety, and depression showed significantly higher ORs in crude analyses, and were thus adjusted for. The multivariate model using these variables led to an adjusted OR of 1.54 (95% CI 0.43-5.31) for HD in relation to PD. Notably, the care time in months was negatively associated with low QoL (adj. OR 0.96; 95% CI 0.93-0.98). Work overload was also associated, but with a higher probability of low QoL (1.04; 1.01-1.08), as was for the presence of anxiety (5.53; 1.71-17.84). The adjusted model fitted well (p=0.34), and explained 49% of the outcome variability.

**Table 4:** Crude and adjusted odds ratios (OR ) with 95% confidence intervals (CI ) from binary logistic regress ion for the probability of low quality of life of caregivers (<70% of the maximum poss ible score resulting from the added normalized scores of the eight dimensions), based on the study on quality of life of caregivers of patients with chronic renal disease in dialysis in northern Mexico, 2019 (n=137)

Variable	Category	OR (95% IC)	
		Crude	Adjusted^3^
Type of dialysis	Peritoneal	1.00	1.00
	Hemodialysis	1.86 (0.79-4.36)	1.51 (0.43-5.31)
Caregivers' age group in years	15-40	1.00	1.00
	41-60	2.86 (0.89-9.17)	2.05 (0.49-8.54)
	>60	4.87 (1.40-16.97)	4.01 (0.87-18.47)
Care time in months	Continuous	0.97 (0.95-0.99)	0.96 (0.93-0.98)
Work overload^1^	Continuous	1.06 (1.03-1.09)	1.04 (1.01-1.08)
Anxiety^2^	No	1.00	1.00
	Yes	10.66 (4.46-25.5)	5.53 (1.71-17.84)
Depression^2^	No	1.00	1.00
	Yes	5.79 (2.61-12.86)	1.07 (0.32-3.60)

1 Based on the Zarit scale score (min 0, max 88): in this sample the minimum value was 0 and maximum value was 71 points.

2 Dichotomized based on the Goldberg's scale: anxiety (≥2/9 items), depression (≥1/9 items).

3 Only statistically significant variables (p<0.05) remained in the final adjusted model; Hosmer & Lemeshow goodness-of-fit test Chi2=8.96 (p=0.34); Nagelkerke R^-^=0.49.

## Discussion

We aimed at comparing the QoL between PCGs of patients in PD and HD from a middle-income city of northern Mexico. Results showed that caregivers in the PD group had better mean scores than those in the HD group in five out the eight dimensions studied. Except for general health, which was significantly higher in the HD group, caregivers in the PD group had a statistically higher means for emotional role functioning, physical functioning, and bodily pain.

Our findings can be compared with the Brazilian study with data from 2003-2006 that included caregivers of elderly patients in HD (n=84), non-elderly patients in HD (n=77), and elderly patients in PD (n=40). The authors reported differences for the physical functioning (p<0.05) and emotional role functioning (p<0.01), dimensions favoring HD caregivers, in contrast with our results that showed better mean scores among PD caregivers. Unlike us, they also found differences for vitality (p<0.05), social functioning (p<0.05), and mental health (p<0.01), but again, favoring HD (19). The other relevant study with 221 Spanish caregivers using data from the early 2000s showed comparable results between caregivers of patients in HD and PD^30^. However, when multivariate adjusted analysis was carried out to predict low QoL (using <70% as cut-off), type of dialysis had no significant impact (adj. OR 1.51; 95% CI 0.43-5.31); the adjusted effect remained non-significant when the cut-off was lowered to <60% (2.11; 0.36-12.3) and <50% (4.71; 0.40-55.5).

Nearly half of the caregivers were middle-aged (41-60 y: 52.5%), and two out of three (78.1%) were married or cohabiting with the patient (51.8%), as others have also noted^19^^,^ 21 ^,^ 27 ^,^ 28 ^,^ 41 ^,^ 42 ^-^ 44.Even though the large majority of PCGs in this study were women (83.9%), as it has been reported extensively^19^^,^ 21 ^,^ 27 ^-^ 29 ^,^ 31 ^,^ 32 ^,^ 41 ^-^ 46, the proportion of male PCGs was particularly higher in the HD (20%) compared with the PD group (7.1%). This finding has not been documented earlier. Apparently, since men are the primary breadwinners, they have to work during the day precluding them to provide the care PD patients require.

No significant differences in the mean or median Zarit burden scores were seen, but PCGs of patients in the HD group had twice the prevalence of medium/severe workload (HD 14.8%, PD 7.2%). These finding replicates what it has been previously reported. In a Turkish study with 127 caregivers, the burden score was significantly higher in the HD group compared with the PD group^32^. Another Turkish study with 114 caregivers also found higher prevalence of intermediate/high burden in caregivers of patients in HD (HD 87%, PD 65%)^31^, and an Indian study of 90 caregivers also reported a higher prevalence of moderate/severe burden in the HD group (HD 40%, PD 23%)^33^. In the adjusted analysis, work overload was associated with a higher probability of low QoL (4% increase risk for each additional point in the Zarit scale) independently of type of dialysis.

According to the Goldberg scale, the prevalence of anxiety (HD 50.5%, PD 19%) and depression (HD 49.5%, PD 16.7%) was much higher in the HD group. These findings can be compared with the Turkish study of 127 caregivers that also showed a lower mean score for anxiety, but a higher mean score for depression in the HD group using the Hospital Anxiety and Depression Scores^32^. In multivariate analysis, only anxiety remained predictive of low QoL among PCGs (i.e., the risk was five times higher), indirectly corroborating the higher levels of anxiety seen in caregivers compared with the general population^47^.

Notably, care time in months was negatively associated with low QoL in multivariate analysis regardless of dialysis type; for each additional month caring for the patient there was a 4% lower chance of having a low QoL. This finding is analogous to that observed in patients with cardiac arrest that showed improvement in caregivers’ wellbeing during the first year associated with adaptive coping styles and resilience^48^; it is possible that PCGs find efficient mechanisms to deal with the physical and emotional burden derived from their care giving activities.

This study had some limitations that ought to be mentioned. One relates to the cross-sectional nature of the study design, as only one assessment of QoL was available precluding relevant longitudinal comparisons. Another limitation relates to the level of patients’ dependency, which can have an impact on the PCGs’ QoL resulting in a possible bias about the significant difference between the comparison groups; while we were unable to assess the dependency level, we adjusted the analyses for the patients’ QoL, measured with the kidney disease QoL short form questionnaire^49^^-^ 50, as a proxy for the dependency level (i.e. the higher the QoL, the lower PCGs’ dependency and vice versa), and found no significant effect (p=0.61), suggesting a non-differential bias. Another limitation relates to the convenience sampling used, which restricts the validity of the results for PCGs in the four hospitals not included, as well as the generalizability of the findings to different settings; yet, the fact that both crude and adjusted analyses led to non-significant differences in PCGs’ QoL among hospitals (p>0.30) points to the possibility of similar findings across hospitals. Lastly, the failure to control for relevant unmeasured factors (e.g. degree of support from other family members), the partial assessment of some variables (e.g. socioeconomic status), and the lack of adjustment due non-statistical significance resulting from a small sample, could have led to residual confounding; in fact, the relatively large confidence intervals observed in the multivariate regression model point to the need for a larger study to better address this topic.

## Conclusion

While PCGs in the PD group had significantly better mean QoL scores for emotional role functioning, physical functioning, and bodily pain, multivariate adjusted analysis showed no differences in the risk of low QoL between PCGz of patients in PD and HD. If these findings are confirmed, they would add to the financial efficiency of the PD modality, and would strengthen its value in resource-limited settings.
